# Conservative endodontic microsurgery to protect critical anatomical structures–selective curettage: a case series

**DOI:** 10.1186/s12903-023-03287-2

**Published:** 2023-08-31

**Authors:** Nan Li, Rui Zhang, Weiwei Qiao, Liuyan Meng

**Affiliations:** 1 The State Key Laboratory of Oral & Maxillofacial Reconstruction and Regeneration, Key Laboratory of Oral Biomedicine Ministry of Education, Hubei Key Laboratory of Stomatology, School & Hospital of Stomatology, Wuhan University, 237 Luoyu Road, Wuhan, 430079 China; 2 Department of Cariology and Endodontics, School and Hospital of Stomatology, Wuhan University, Wuhan, China

**Keywords:** Endodontic microsurgery, Mandibular canal, Nasopalatine nerve tube, Selective curettage, Vital teeth.

## Abstract

**Background:**

Endodontic microsurgery has yielded highly successful outcomes in preserving teeth with persistent or recurrent cases of periapical periodontitis that could not be successfully treated by nonsurgical endodontic approaches. To avoid complications in conditions in which periapical lesions invade anatomical structures such as the nasopalatine nerve tube and mandibular canal, selective curettage has been proposed as an alternative choice of complete curettage in surgery.

**Case presentation:**

The 8 cases reported herein had undergone root canal treatment and/or retreatment but still presented with symptoms, such as recurring sinus tracts and persistent dull pain. The radiographic examination indicated a large area of radiolucency that was associated with the tooth and had invaded adjacent critical anatomical structures. The patients opted for selective curettage via endodontic microsurgery, and the lesions were histologically confirmed as periapical cysts or granulomas. The follow-up results for one year or more indicated that the affected teeth were clinically asymptomatic and exhibited complete or incomplete healing radiographically.

**Conclusion:**

This case series provides clinical evidence for the feasibility of selective curettage in endodontic microsurgery, which can avoid complications caused by damage to the adjacent critical anatomical structures.

## Introduction

Endodontic microsurgery (EMS) is a treatment option for teeth that have failed root canal treatment (RCT) or retreatment (ReRCT) and/or have severe anatomical variations [1]. Curettage, namely, removing pathological periapical tissues or foreign materials beyond the apical foremen in surgery, controls sources of infection and facilitates retrograde preparation and filling [2]. Periapical diseased tissues are generally curetted completely [3]. However, there are some cases in which the lesions intrude upon anatomical structures, such as the mandibular canal and adjacent vital teeth, which pose great challenges to complete curettage. Selective curettage has thus been proposed as an alternative choice for these cases, to preserve contiguous vital structures. As early as 1996, Lin et al proposed that there was no need to curette all the inflamed periapical tissues during surgery because the composition of inflammatory tissues was similar to that of healing granulation and inflammation may be part of the healing process [2]. Nesari et al also put selective curettage into practice in 2020 and reported a satisfying prognosis [4]. However, evidence regarding whether inflamed tissues should be completely curetted and whether remaining tissues have an effect on prognosis is still very limited. In addition, all reported cases were confirmed as periapical granulomas by biopsy, and no cases of periapical cysts have been reported thus far.

The aim of this case series was to provide more clinical evidence for the feasibility of selective curettage [4] in EMS, which provided an alternative approach for lesions adjacent to critical anatomic structures to avoid complications. Regular clinical and radiographic examinations were performed to assess the healing status with follow-up periods ranging from 12 to 66 months.

## Materials and methods

We retrospectively evaluated the outcomes of 10 teeth from 10 patients who had received conservative endodontic microsurgery. The inclusion criterion was that the teeth had been previously treated before surgery, but the symptoms were persistent or recurrent. The radiographic examination indicated that the lesions were close to the critical structures, which posed a great challenge to complete curettage. Informed consent was obtained. Preoperative examinations (blood coagulation function examination, infectious disease examination, periapical radiography (PAR) and cone-beam computed tomography (CBCT (Morita, J. Morita MFG. CORP, Kyoto, Japan)) were taken before EMS. All the patients denied a contributory and allergic history. Surgeries were performed by two experienced endodontists. The surgical procedures were performed under an endodontic microscope (OPMI PICO; Carl Zeiss, Gottingen, Germany). Patients were given local anaesthesia (STA single tooth anaesthesia system (Milestone Scientific, Livingston, NJ)) with 4% articaine hydrochloride with epinephrine (1:100,000). Then, intrasulcular incisions were made and the full-thickness flap was elevated. After locating the root apex, an osteotomy was created, followed by root resection with a handpiece in the anterior area or an angled handpiece in the posterior area. Selective curettage, which removed approximately 40–70% of the lesions, was performed using a curette with sterile saline irrigation. The curetted tissues were immediately fixed with paraformaldehyde solution and then sent for histopathological examination. The resected root surface was stained with methylene blue and inspected carefully under high magnification. Retrograde preparation was performed with ultrasonic tips along the long axis of the tooth and retrograde filling with iRoot BP Plus (Innovative Bioceramics, Vancouver, BC, Canada). Guided tissue regeneration (GTR) with graft materials (Geistlich Pharma Ag, Switzerland) and a bioactive membrane (Geistlich Pharma Ag, Switzerland) was conducted for two cases with large lesions or through lesions [5, 6]. The flap was repositioned and sutured with monofilament sutures at the end of surgery. No antibiotics were prescribed, except for patients who underwent GTR. Ibuprofen (0.2–0.4 g) every 4–6 hours was recommended for pain relief. Sutures were removed 5–7 days after the procedure, and the cases were followed up clinically and radiographically for at least 12 months. The outcome of microsurgery was categorized using clinical and radiographic criteria (Molven’s criteria [7] or modified Penn 3D CBCT criteria [8, 9]). Detailed clinical and radiographic characteristics of the cases are summarized in Table 1.

### Case presentation

#### Case 1: tooth #8, adjacent to the nasal fossa and involving tooth #7 and tooth #9

**Fig. 1:**
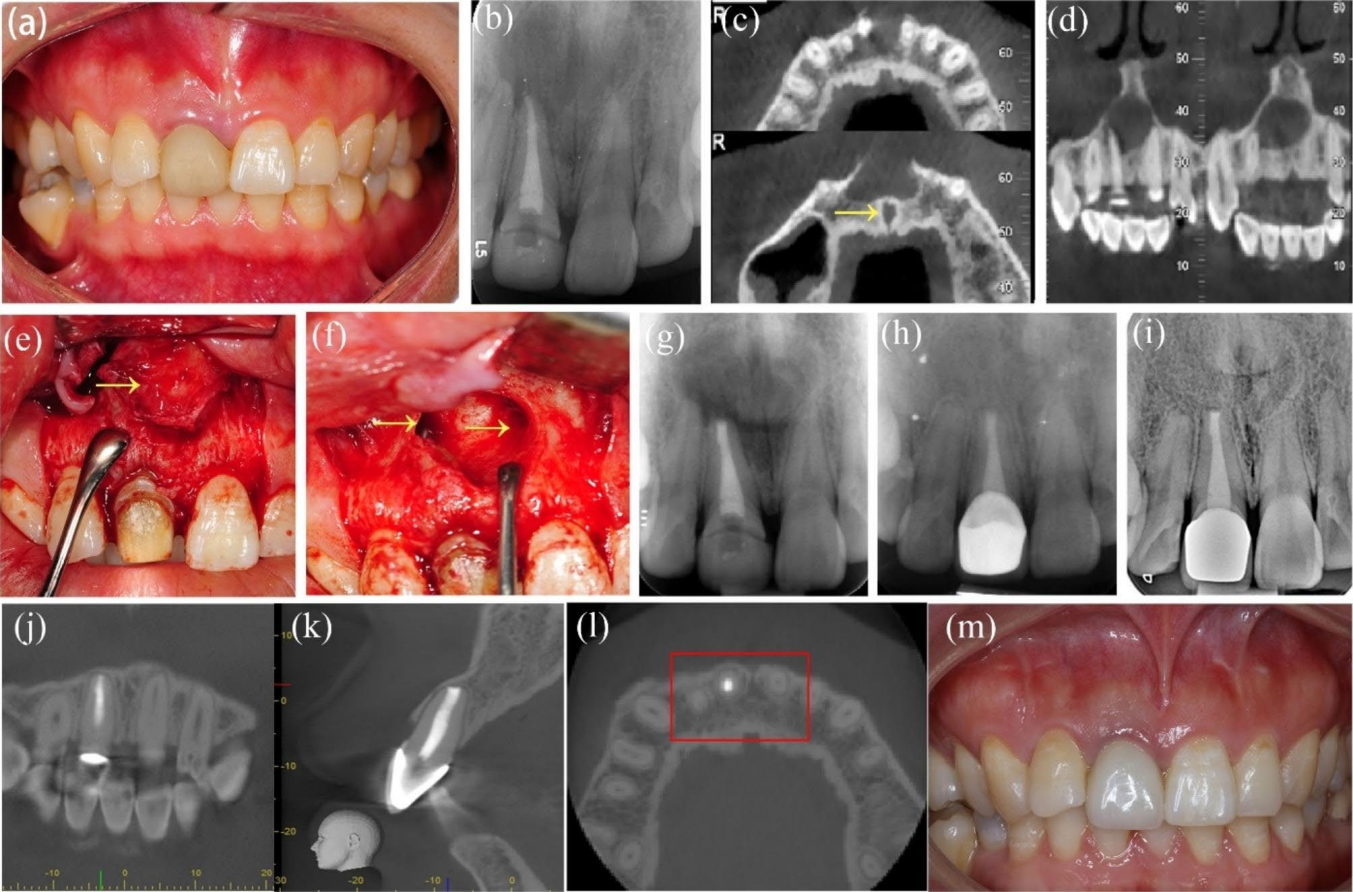
**-** Case 1: tooth #8, adjacent to nasal fossa and involving tooth #7 and tooth #9. (***a****)* The initial intraoral photograph showing the swollen gingiva. (***b****)* The initial PAR showing a large lesion of tooth #7, #8 and #9. (***c****-****d***) The preoperative CBCT axial and coronal view slices showing involvement of tooth #7 and tooth #9 and the proximity of the lesion to the nasopalatine nerve tube and nasal floor (The arrow points to nasal fossa). (***e****-****f****)* The intra-operative photograph before and after curettage; the arrows indicating the granulation tissues (left) and the nasal floor (right). (***g***) The postoperative PAR of EMS of tooth #8. (***h****)* 30-month follow-up PAR showing complete healing. (***i****-****m***) 60-month follow-up PAR, CBCT coronal, and axial view slices showing limited healing and intraoral photograph

A 27-year-old woman presented in our department for gingival swelling in the maxillary right anterior area for 6 months. She had received RCT of tooth #8 several years previously and ReRCT 6 months prior because of swollen gingiva. Upon clinical examination, tooth #8 had a provisional crown with palpation tenderness, no cold response, and no percussion tenderness, and the probing depth was within the normal limits. PAR and CBCT revealed that tooth #8 had been previously treated with a large periapical radiolucency extending from tooth #7 to tooth #9; tooth #7 and #tooth 9 were responsive to cold and were within normal limits in sensitivity tests. Tooth #8 was diagnosed with periapical periodontitis, and EMS for tooth #8 was performed. Lesions around tooth #8 were thoroughly curetted, but those close to tooth #7, #9 and the nasal fossa were untouched. The pathological report indicated a periapical cyst. The patient remained asymptomatic after 66 months. The follow-up PAR showed complete healing, and CBCT indicated limited healing for the discontinuity of the cortical plate (Fig. 1a-m).

#### Case 2: tooth #29, with involvement of tooth #28 and the mesial root of tooth #30

**Fig. 2:**
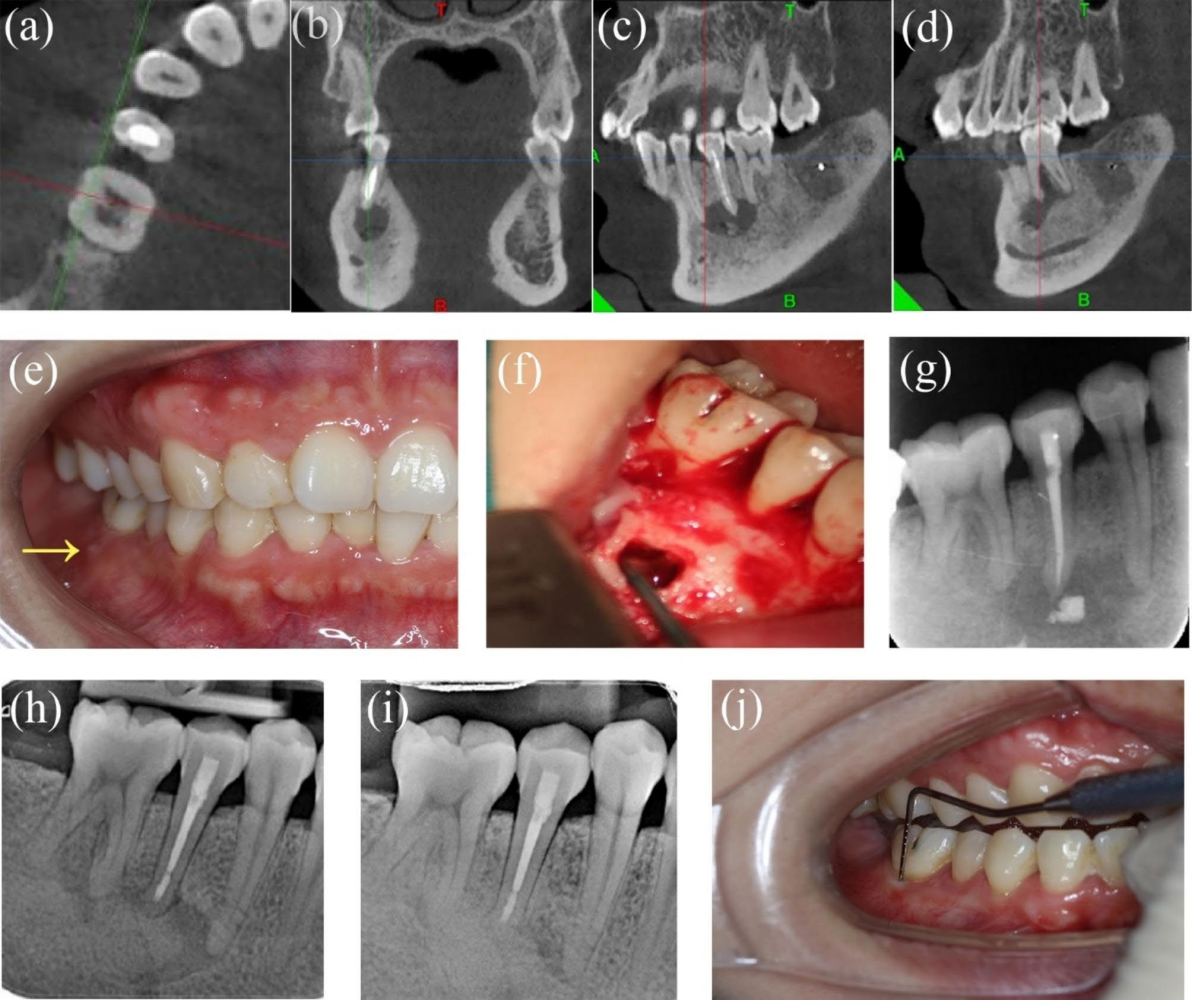
**-** Case 2: tooth #29, involvement of tooth #28 and the mesial root of tooth #30. (***a****-****d***) The preoperative CBCT axial, coronal and sagittal view slices showing the involvement of tooth #28 and the mesial root and furcation of tooth #30. (***e***) Pre-operative photograph; the arrow showing the sinus tract. (***f***) Intra-operative photograph of curettage. (***g***) Preoperative PAR. (***h***) Postoperative PAR after EMS and GTR. (***i****-****j****)* 12-month follow-up PAR revealed incomplete healing and intraoral photograph

The patient was a 35-year-old woman whose chief complaint was swollen gums on the buccal side of the mandibular right first molar, recurrently for 1 year. Tooth #29 had undergone RCT in a local hospital 2 years prior. Tooth #29 had no cold response and no percussion or palpation tenderness. Periodontal probing was within normal limits. Tooth #30 had a sinus tract on the buccal side and responded normally to cold and electric pulp tests. PAR and CBCT revealed a previously treated root canal of tooth #29, and there was an 8 × 14 × 15 mm radiolucent area around the apical area of teeth #28, #29. The radiolucency penetrated through the apical area to the furcation area of tooth #30. Teeth #28 and #30 were both within normal limits for cold and electric pulp tests. Tooth #29 was diagnosed with periapical periodontitis and EMS for tooth #29 was planned. After flap elevation and osteotomy of tooth #29, selective curettage of 50% of the lesion was performed, leaving those adjacent to teeth #28 and #30. GTR was performed for tooth #29 and the furcation of tooth #30 to promote primary healing of the large bone defect. The pathological report indicated a periapical granuloma. Tooth #28 and tooth #30 were tested as vital pulp after 12 months, and PAR indicated incomplete healing of tooth #29 (Fig. 2a-j). The patient refused a CBCT follow-up considering radiation concerns.

#### Case 3: tooth #9, with a large through-and-through lesion and involvement of the nasopalatine nerve tube and tooth #10

**Fig. 3:**
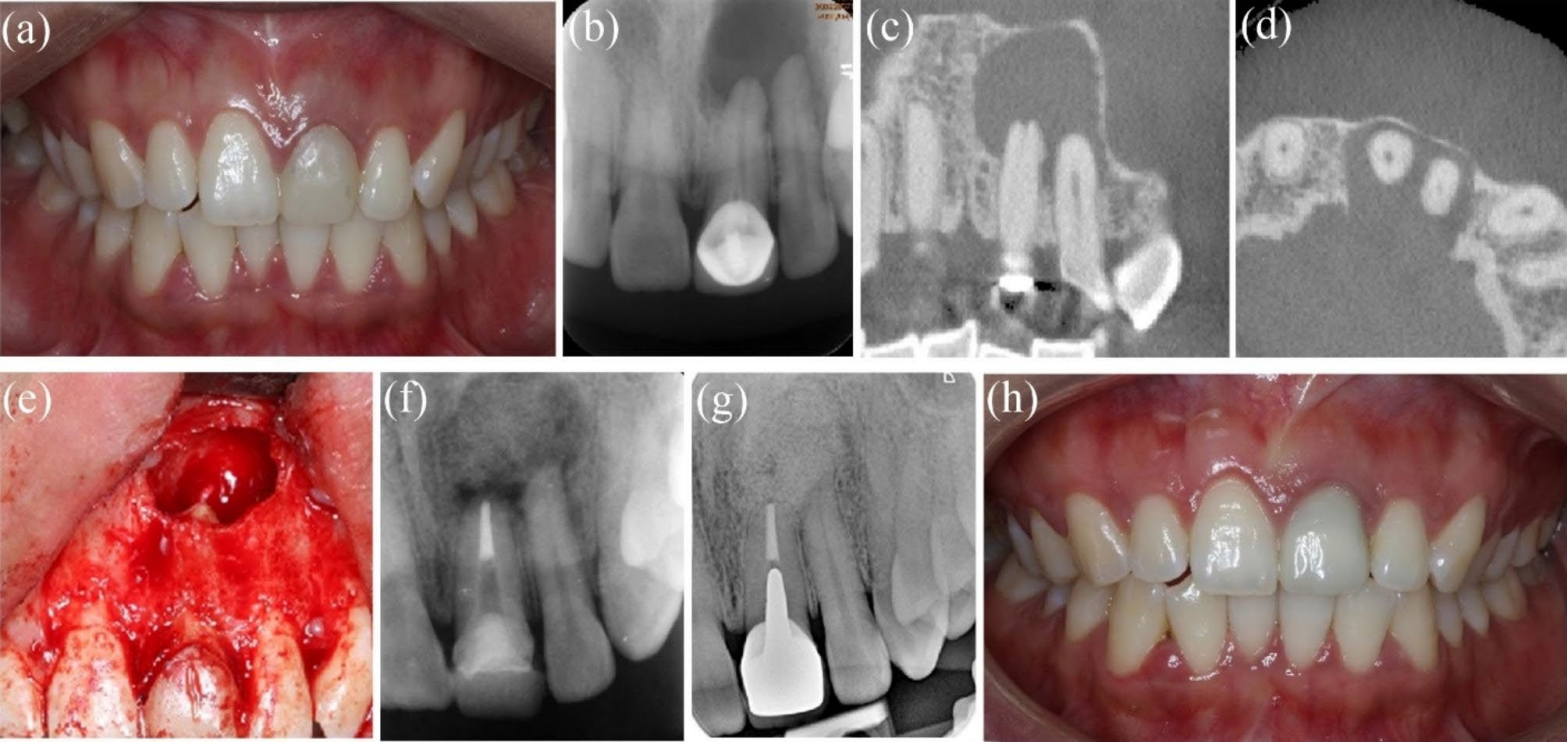
**-** Case 3: tooth #9, involvement of nasopalatine nerve tube and tooth #10. (***a***) Pre-operative intraoral photograph. (***b****-****d***) Preoperative PAR and CBCT indicating lesions involved nasopalatine nerve tube and tooth #10. (***e***) Intraoperative photograph after curettage. (***f***) Postoperative PAR of EMS and GTR.(***g****-****h****)* 27-month follow-up PAR and photograph indicating complete healing

A 22-year-old woman was referred to us for gum swelling in the maxillary left anterior area for several days. There was an all-ceramic crown restoration of tooth #9. The examined area demonstrated a sinus tract on the palatal mucosa and a normal periodontal probing depth around the tooth. Tooth #9 was painful to percussion and had no response to the cold test. Thermal and electric vitality test results were positive in tooth #10. PAR and CBCT showed a large area of periapical radiolucency (approximately 15 × 10 × 15 mm) involving tooth #9 and tooth #10 as well as the nasopalatine nerve tube. Tooth #9 was diagnosed with periapical periodontitis. RCT was then performed for tooth #9, and regular follow-ups were advised for tooth #10 to assess its pulp vitality. Due to the persistent purulent drainage of tooth #9 in the following 3 months, EMS, as well as GTR, was recommended for the large through-and-through lesions in the apex. Lesions were selectively removed to preserve the nasopalatine nerve tube and vitalization of tooth #10. Histopathology examination reported a periapical cyst. At the 27-month follow-up, the patient did not report any clinical symptoms, and PAR indicated complete healing (Fig. 3a-h).

#### Case 4: tooth #29, adjacent to the mandibular canal and mesial root of tooth #30

**Fig. 4:**
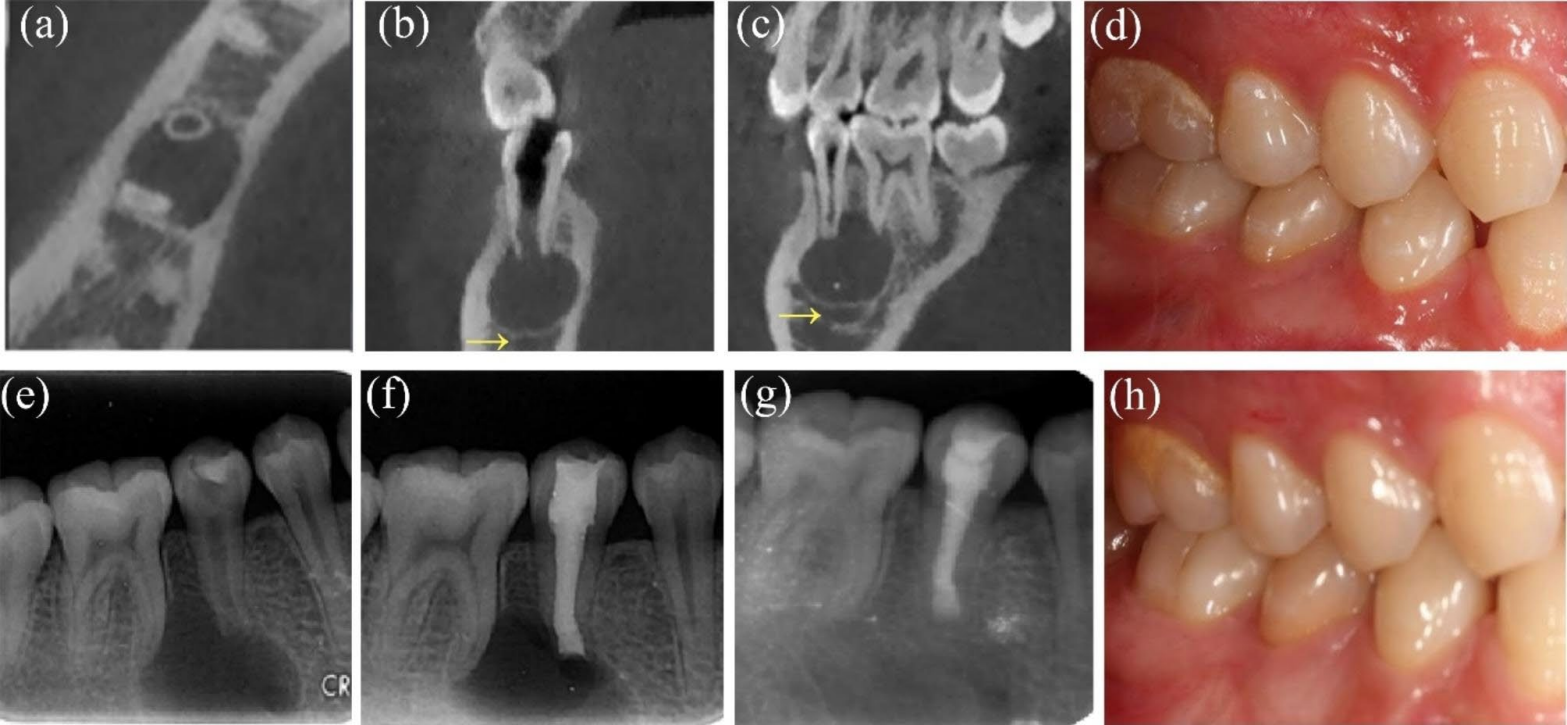
**-** Case4: tooth #29, adjacent to mandibular canal and mesial root of tooth #30. (***a****-****c***) The preoperative CBCT sagittal, axial and coronal views showing proximity to the mandibular canal (arrows) and the mesial root of tooth #30. (***d***) Preoperative intraoral photograph. (***e***) Preoperative PAR showing a lesion on tooth #29 and the mesial root of tooth #30. (***f***) Postoperative PAR of EMS. (***g****-****h***) 12-month follow-up PAR and photograph showing complete healing

A 21-year-old woman came to our department with complaint of gingival swelling and occlusal pain in the mandibular right posterior area for several days. The patient reported that tooth #29 had undergone RCT five years before and unfinished ReRCT had been performed at another hospital several days prior for occlusal pain. On examination, tooth #29 had a large access cavity on the occlusal surface with a cotton pellet and abundant pus and blood secretions. The buccal mucosa near tooth #29 was red and swollen, with a sense of fluctuation and pain on palpation. No response to thermal challenge and no pain on percussion were demonstrated, and all probing depths were less than 3 mm. The preoperative PAR and CBCT indicated that there was a large-area apical radiolucency encompassing tooth #29 and the mesial root of tooth #30 and proximity to the mandibular canal. However, tooth #30 had no clinical symptoms and a normal response to the electric pulp test. Therefore, tooth #29 was diagnosed with periapical periodontitis and ReRCT for tooth #29 was performed, but the patient complained of gingival swelling every time after medication and temporization. Consequently, EMS was suggested for tooth #29. To protect the mandibular canal and avoid devitalization of tooth #30, only 50% of the lesion just around tooth #29 was removed. The biopsy report confirmed a periapical cyst. No clinical symptoms were observed in tooth #30, and the 12-month follow-up PAR indicated complete bone healing of tooth #29 (Fig. 4a-h).

#### Case 5: tooth #9, with involvement of tooth #10 and penetration of the labial cortical bone

**Fig. 5:**
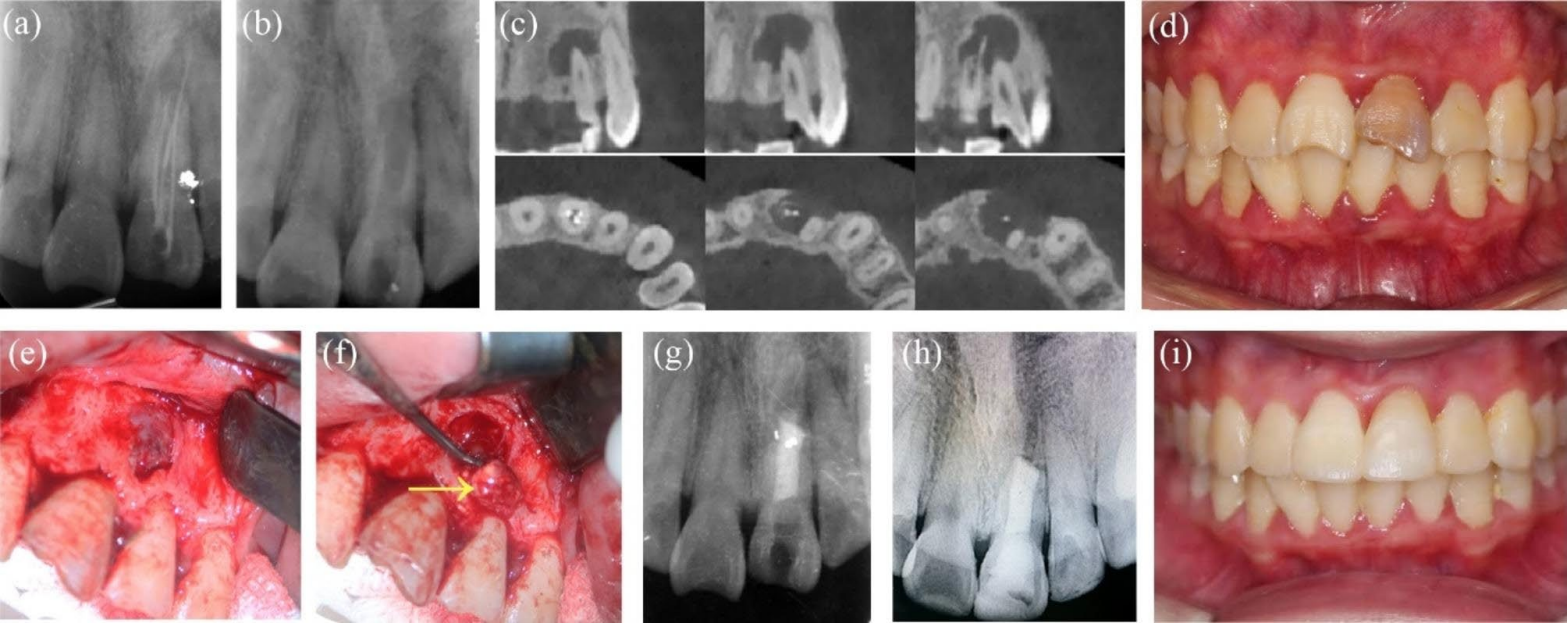
**–** Case5: tooth #9, tooth #10 involved and penetration of labial cortical bone. (***a***) The initial PAR showing the lesion on tooth #9 and #10. (***b***) Pre-operative PAR after 6-month medication. (c) Preoperative CBCT showing the large defect of labial cortical bone. (d) Preoperative intraoral photograph. (***e***) Incision and elevation. (***f***) Cyst (arrow) excision. (*G*) Postoperative PAR of EMS and obturation. (***h****-****i****)* 30-month follow-up PAR and photograph showing complete healing

A 22-year-old woman presented with tooth discolouration and defection in the maxillary anterior area for 10 years. The patient reported a history of trauma and RCT of tooth #9 10 years prior. Clinical examination revealed that the incisor edge of tooth #9 was defective, and the tooth surfaces showed brownish yellow discolouration. An access cavity could be seen on the lingual surface of tooth #9, with discomfort to percussion, physiologic mobility and no palpation tenderness. Tooth #9 and tooth #10 were both involved in the periapical radiolucency revealed by PAR and CBCT, while tooth #10 had no clinical symptoms. Tooth #9 was diagnosed with periapical periodontitis, and ReRCT was planned for tooth #9. However, the exudation in the root canal remained uncontrolled after we had tried all the nonsurgical treatment methods and 7 months’ regular intracanal medication, so EMS was suggested. To avoid devitalization of tooth #10, approximately 40% of the lesion was selectively curetted without touching the distal apical tissues of tooth #10. The biopsy indicated a periapical cyst. The patient reported no symptoms after 30 months of follow-up, and PAR revealed complete healing (Fig. 5a-i).

#### Case 6: tooth #8, with involvement of tooth #7

**Fig. 6:**
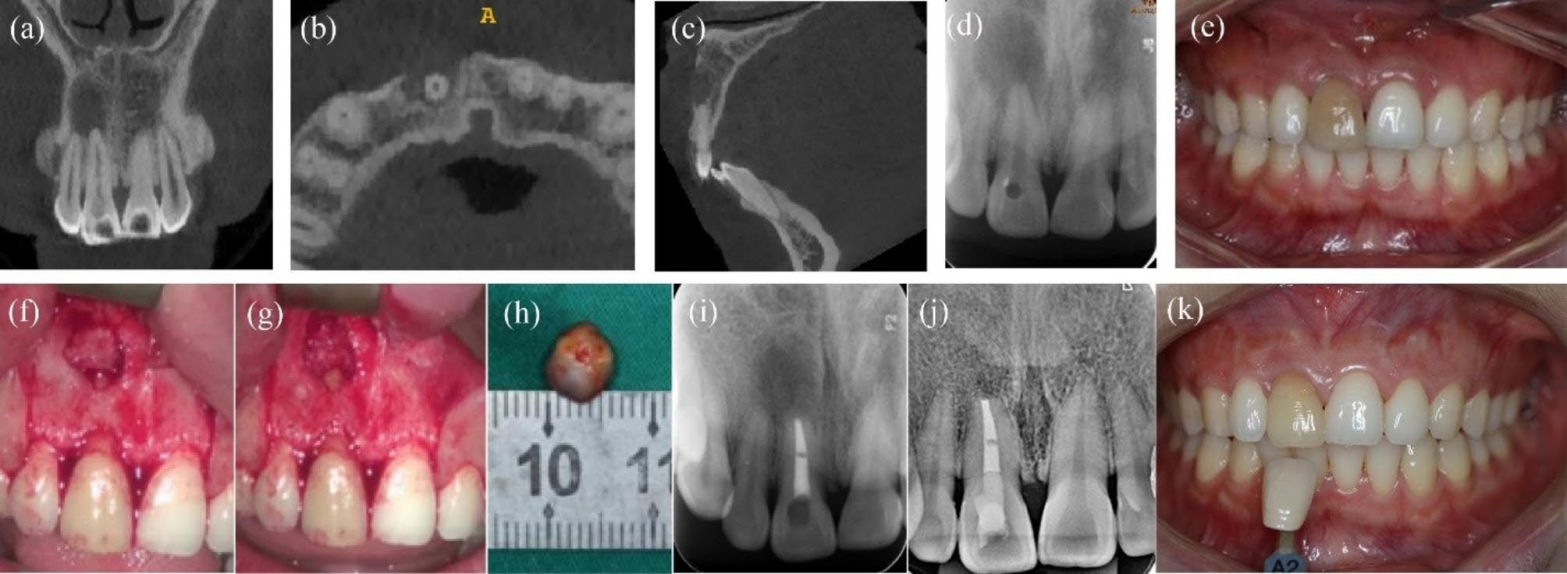
**-** Case 6: tooth #8, involvement of tooth #7. (***a****-****c***) The preoperative CBCT coronal, axial and sagittal views showing involvement of tooth #7. (***d****-****e***) Preoperative PAR and intraoral photograph. (***f****-****g***) Before and after enucleation of cyst. (*h)* The picture of cyst. (*I*)Postoperative X- ray of EMS and obturation. (***j****-****k***) 44-month follow-up PAR showing complete healing and photograph (after bleaching)

The patient was a 30-year-old woman who was referred for recurrent swelling in the maxillary right anterior teeth for several months. Tooth #8 had been treated in another hospital 1 month prior, but the patient was still symptomatic. Tooth #8 was discoloured, and there was an access cavity on the lingual tooth surface. Tooth #8 showed slight tenderness to percussion, had normal probing depths and no response to thermal testing. The preoperative PAR and CBCT showed a periapical radiolucency extending from the apex of tooth #8 to tooth #7. Tooth #7 was responsive within normal limits to cold and electric pulp tests. ReRCT was performed for tooth #8, but the intracanal exudation persisted for more than 3 months. EMS or decompression with placement of a tube to maintain drainage was suggested. The patient consented to undergo microsurgery. The cyst around the apex of tooth #8 was enucleated in whole, and the surrounding tissues near the apex of tooth #7 were untouched. Periapical cysts were confirmed by histopathology examination. The 44-month follow-up PAR indicated complete healing. Tooth #7 maintained pulp vitality according to cold and electric pulp tests and had no clinical symptoms (Fig. 6a-k).

#### Case 7: tooth #29, with involvement of the mesial root and furcation of tooth #30

**Fig. 7:**
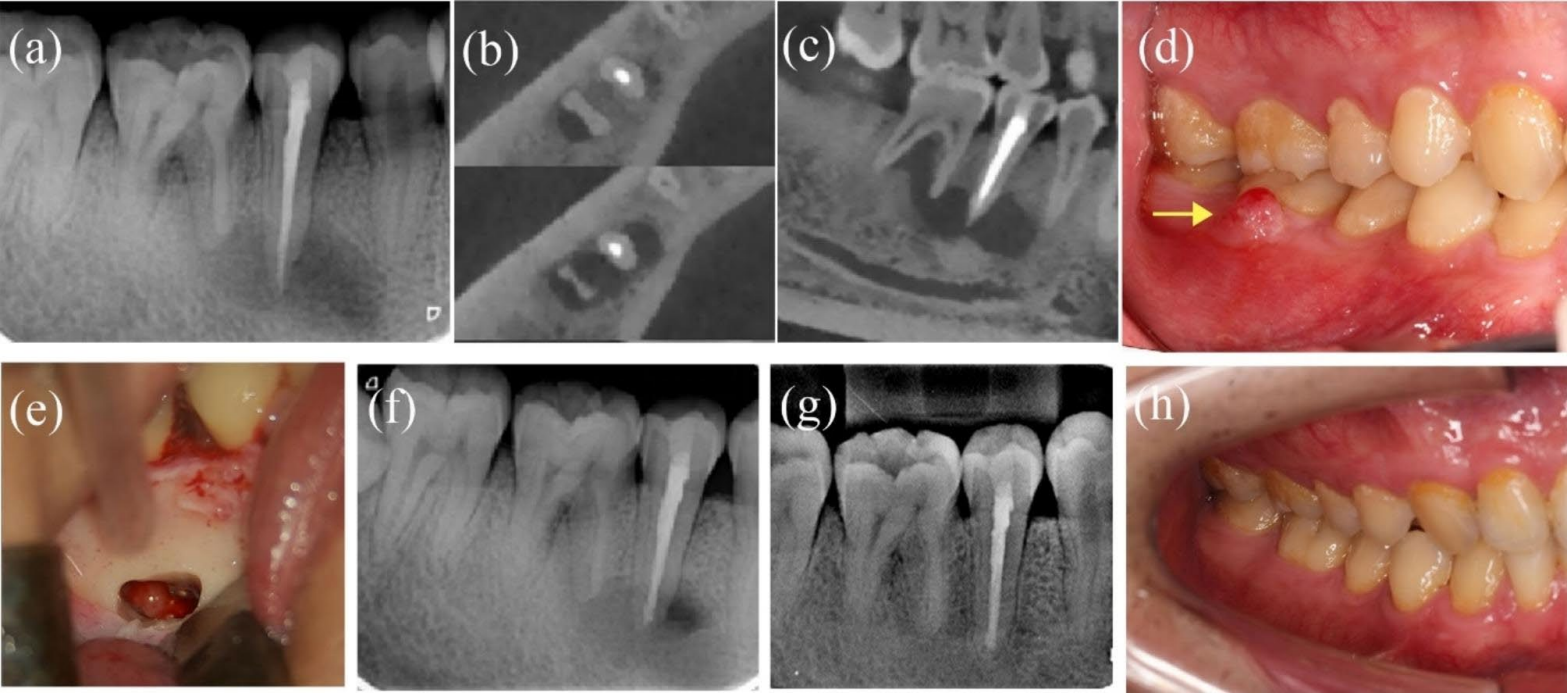
**–** Case7: tooth #29, the mesial root and furcation of tooth #30 involved. (***a***) Preoperative PAR indicating lesions on tooth #29 and tooth #30. (***b****-****c****)* Preoperative CBCT indicating the involvement of the mesial root and furcation of tooth #30. (***d***) Preoperative intraoral photograph; the arrow indicating the sinus tract. (***e***) A clinical picture of osteotomy size. (f) Postoperative PAR of EMS. (***g****-****h***) 24-month follow-up PAR and photograph showing complete healing

A 25-year-old man presented with dull pain in the right posterior mandibular area for 6 months. Tooth #29 had filling material on the occlusal surface and was sensitive to percussion; the tooth had undergone RCT five years before. Tooth #29 was tender to percussion, had negative results upon thermal testing and exhibited normal probing depths. A sinus tract was detected on the buccal gingiva of tooth #30. The probing or percussing test was negative, and the electric pulp test showed a vital pulp. PAR and CBCT revealed a large radiolucent area around the apex of tooth #29 and the mesial root and furcation of tooth #30. The option of nonsurgical ReRCT and EMS for tooth #29 was given to the patient, and the advantages and disadvantages were explained. The patient chose surgical intervention. Tissues proximity to the mesial root of tooth #30 remained untouched. At the 24-month follow-up, tooth #29 and tooth #30 had no clinical signs or symptoms, and PAR indicated complete healing (Fig. 7a-h).

#### Case 8: tooth #19, with involvement of the mandibular canal

**Fig. 8:**
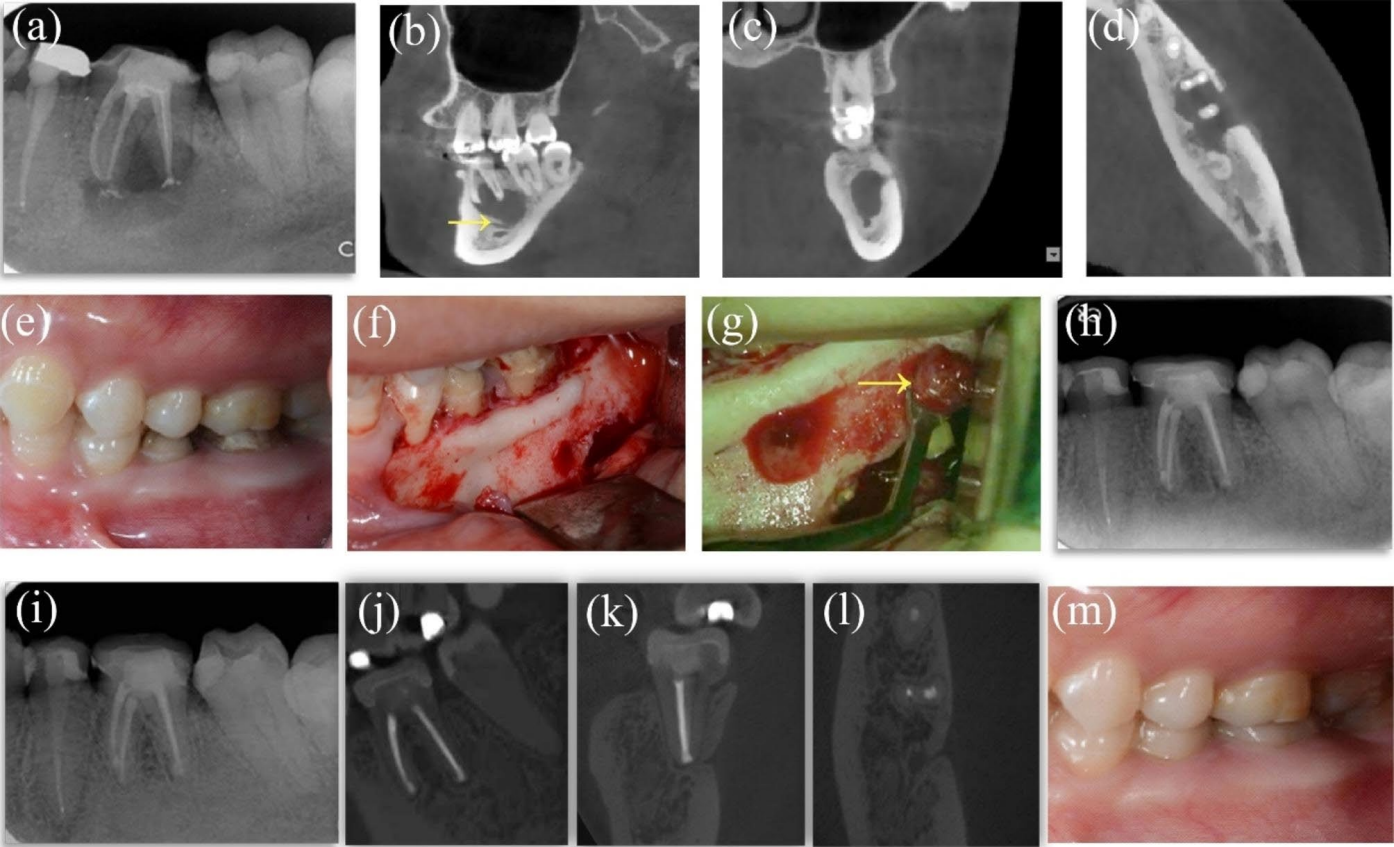
Case8: tooth #19, the mandibular canal was involved. (***a***) Preoperative PAR revealing the radiolucency of tooth #19. (***b****-****d***) Preoperative CBCT of coronal, axial and sagittal views indicating the involvement of mandibular canal (arrows) and buccal cortical bone. (***e****)* Preoperative intraoral photograph. (***f****-****g***) Flap elevation and granulation enucleation; the arrow indicating the granulation. (***h***) Postoperative PAR of EMS. (***i****-****m***) 48-month follow-up PAR showing complete healing and CBCT showing limited healing and photograph

A 33-year-old woman complained of swelling on the buccal gingiva of the left lower posterior tooth for ten days; she had received RCT in a local hospital 4 years ago. Clinical examination revealed that the crowns of tooth #19 had been removed. Tooth #19 was filled with black materials on the distal occlusal surfaces, accompanied by no response to the thermal test and no pain to percussion. The periodontal examination was within normal limits. There was a large periapical radiolucency of tooth #19 on the PAR. Tooth #19 was diagnosed with periapical periodontitis. The patient still complained of sustained tenderness to percussion after the following ReRCT, so the EMS for #19 was planned. Preoperative CBCT revealed a lesion of 18 × 11 × 11 mm around the apex of #19; the lesion extended to the mandibular canal. To avoid damaging the mandibular canal, approximately 70% of the lesion around the root tip of tooth #19 was curetted, and the others next to the mandibular canal remained. The 48-month PAR follow-up indicated complete healing of tooth #19 with no clinical symptoms, while CBCT showed limited healing (Fig. 8a-m).

## Discussion

The main causes of posttreatment periapical periodontitis (persistent, emergent or recurrent) are categorized as persistent intraradicular infection, extraradicular infection, foreign body reactions of exogenous materials, endogenous cholesterol crystals, true cysts and fibrous scar tissues [10–13]. To preserve teeth with persistent periapical periodontitis that have been endodontically treated, orthograde revision should be considered first to eliminate bacteria remaining in the root canal system and to achieve complete filling. When nonsurgical endodontic treatment or retreatment is not feasible, surgical measures [12] (EMS, intentional replantation, autotransplantation of teeth or exaction) should be applied.

EMS obtained a high success rate in the present literature, which ranged from 69.3 to 93.3% in a meta-analysis of 10 clinical studies followed up for 2–13 years [14] with endodontic microscopy, microsurgical instruments, ultrasonic tips and bioceramic materials [15, 16]. Floratos et al proposed that nearly all teeth with endodontic lesions can be successfully treated by careful patient selection [17], and Kim et al found a successful outcome of 95.2% for cases that were purely endodontic lesions after EMS, while the success rate was only 77.5% for cases associated with periodontal diseases [18].

The aim of curettage in EMS is to remove diseased periapical tissues, foreign bodies or root fragments in the apex area, while the selective curettage means to selectively maintain the inflammatory tissues close to the mandibular canal, maxillary sinus or adjacent vital pulp teeth to protect anatomical structures and avoid complications. Fish histopathologically divided periapical periodontitis into 4 zones, namely, infected areas (zone 1 and zone 2), containing bacteria and bacterial toxins ans byproducts; and zone 3 and zone 4, which were filled with products of irritants and components of healing granulation tissues [19]. It was reported that infectious microorganisms are mainly deposited on the surface of the root tip, in the necrotic periapical tissues adjacent to the apical foramen, or in the body of the lesion, and peripheral inflammation is simply a response to bacterial toxins or harmful metabolic byproducts [2]. Therefore, it might be reasonable to postulate that the lesions could be eliminated partially because peripheral inflammatory tissues get into resolution with the removal of irritants, and those granulation-like tissues are incorporated into new granulation tissues as part of the healing process [2].

Nesari put selective curettage into practice by removing approximately 50-70% of the granulomatous tissue in cases that had large through-and-through lesions, perforating into the nasal fossa, or in proximity to the mandibular canal [4]. All the cases reported showed complete or incomplete healing in PAR and CBCT images at follow-ups and were confirmed as granulomas by biopsy [4]. Three cases of periapical granulomas in our report showed the same prognosis as Nesari’s. However, few cases of selective curettage have been reported thus far when the biopsy report indicates periapical cysts.

According to Carrillo et al, the prognosis of periapical lesions differs with size and pathological results, and large lesions and periapical cysts tend to have worse prognosis than others [20]. Apical periodontitis is actually the host defence response to infection of the root canal system [21], and periapical cysts are formed by the proliferation of Malassez epithelial cells under the stimulation of inflammatory mediators [22], whose incidence ranges from 6 to 50.45% [23–28]. Two kinds of periapical cysts (true cysts and pocket cysts) were reported to have incidences of 9% (true cysts) and 6% (pocket cysts), respectively [27]. Pocket cysts have a direct connection with the root canal through the apical foramen, while true cysts are independent to the root canal [28]. True cysts are believed to persist after nonsurgical treatment, while pocket cysts can heal after RCT (eliminating infectious agents of the root canal and preventing reinfection by obturation) [21, 29, 30]. Several studies have been working on the differentiation of periapical granulomas and cysts preoperatively by CBCT and magnetic resonance imaging (MRI), suggesting the feasibility of pretreatment noninvasive diagnostic methods [31–34]. However, there are no effective ways to distinguish true cysts from pocket cysts except by collecting surgical samples for biopsy using a serial sectioning approach, so it is nearly impossible to make corresponding treatment plans preoperatively in light of the different pathological types. Ricucci et al assessed the clinical manifestations and radiographic and histologic features of the two kinds of cysts and found no significant differences, concluding that there was no need to differentiate them, both lesions were related to intra- and extraradicular infections [28]. Extraradicular bacteria were found in some cases either inside the cyst lumen or as a biofilm adhered to the outer root surface near the exits of the apical foramina, which could not be eliminated by nonsurgical root canal procedures [28]. The histobacteriologic results also confirmed the same aetiology of true cysts as pocket cysts, such as intraradicular infections or concurrent extraradicular infection [12, 28, 35], suggesting that true cysts may not be the direct cause of failure of RCT but of uncontrolled or persistent infection.

Recently, several cases with large periapical cystic lesions adjacent to critical anatomic structures were treated successfully with decompression and nonsurgical endodontic treatment [35, 36]. Together with eliminating bacteria from the root canal, draining cystic fluid containing bacterial colonies and cholesterol crystals and disrupting the epithelial lining could lead to resolving larger cyst-like periapical lesions. The drawbacks of decompression might include the long-term treatment procedure, patient compliance, and possible infection of the exposed cavity; if decompression fails, a subsequent surgical intervention should be performed. Therefore, it is supposed that when preoperative examinations indicate that a periapical cyst is in close proximity to anatomical structures, selective curettage of the infective tissues and destruction of the epithelial lining can be suggested as an effective alternative treatment option with thorough RCT plus removal of the periapical 3 mm end of the root and filling [10].

The histology of Nair’s study revealed that nearly two-thirds of the lumen was occupied by necrotic tissue, containing cells at different stages, and the narrow band of connective tissue surrounding and enclosing the cyst cavity was almost free of inflammation [30]. Bhaskar also proposed that periapical cysts could be converted into granulation to facilitate resolution by mechanically destroying or disrupting the epithelium lining of cysts [37], which was also performed in selective curettage. Several studies also confirmed that rupturing the cyst sac could promote the healing of large cysts, although eliminating part of the lesions [11, 25]. Some case reports provided favourable clinical evidence as well [36, 38]. In addition, the healing process of cysts actually involves the apoptosis of endothelial cells, inflammatory cells and fibroblasts. Once the irritants dissolve, those cells gradually undergo programmed cell death and do not release proinflammatory factors, although there are some remnants in the apex [39]. Our cases (cases 1, 3, 4, 5 and 6) presented above were mostly confirmed to be periapical cysts by biopsy, which all underwent nonsurgical treatment (RCT and ReRCT) preoperatively, but periapical periodontitis or purulent drainage persisted, so EMS was performed. Owing to these lesions involving critical anatomical structures, we adopted the method of completely removing the apical infected tissues of the root by selectively curetting peripheral inflammatory tissues and destroying the epithelial lining of cysts to promote healing as well as avoid complications.

Taschieri et al reported a significantly higher success rate of GTR in penetrating lesions, but no significant difference was observed in cases that did not affect the buccal and lingual cortical bone [6, 40]. It was also shown that there was no correlation between the success and survival rate of endodontic microsurgery and the application of GTR, but a significant correlation between the application of GTR and the quality of apical bone remodelling was observed in CBCT images [41].

In the present study, GTR was applied in cases 2 and 3, which were both large periapical lesions (bone defects larger than 10 mm [20]). Case 2 had furcation involvement, and case 3 involved palatal cortical bone. Over 1 year of follow-up, PAR showed complete 2D healing in both cases.

The healing assessment was based on Molven’s 2D criterion and Penn 3D CBCT criteria. For 2D examination, the lesion can only be visible when the periapical radiolucency reaches nearly 30–50% of bone mineral loss and lesions confined within the cancellous bone cannot be detected. A 2D lesion area addresses the mesiodistal and apicocoronal extent of the defect, while buccolingual directions on average have the largest extent of a periradicular defect, which may lead to misjudgment. As a three-dimensional imaging technology, CBCT can assess the extent and location of periapical lesions, the bone thickness and the proximity to anatomic structures such as the mandibular canal, nasopalatine nerve tube or adjacent vital teeth. In addition, CBCT can also be used to evaluate odontogenic versus nonodontogenic symptoms, and the good reproducibility and repeatability of CBCT allow us to precisely assess the stage of healing, which is difficult to discern in 2D examination. Treatment outcomes were categorized as complete healing, limited healing, uncertain healing and unsatisfactory healing [9]. The absence of clinical symptoms plus complete healing found by PAR or CBCT were undoubtedly considered success [7, 8]. PAR or CBCT indicating incomplete healing or scar tissue at the 1-year follow-up after surgery can also be regarded as success [42, 43] because they would develop into complete healing in the following years or just retain scar tissues, which is confirmed by long-term follow-up [42]. In our report, the outcome of all cases was assessed as successful with no adverse effects.

A limitation of our study was the fact that the age of the included patients was randomly selected (ranging from 21 to 36 years). According to the literature, most systematic reviews and meta-analyses have shown no statistically significant differences between patient age and the prognosis of endodontic microsurgery [14, 44, 45], while some studies have suggested that increasing age has a negative impact on the prognosis of apical surgery [46]. Two patients over 40 years were also assessed as having complete healing after microsurgical sparing, which was reported by Nesari in 2020.

In conclusion, this case series provided clinical supportive evidence for selective curettage in EMS. It can be considered an alternative strategy to avoid complications in cases of periapical granulomas or cysts involving critical anatomical structures. However, if the lesion shows uncertain or unsatisfactory healing at the 1-year follow-up and/or the symptoms persist, the patient must be informed of the possibility of resurgery; alternatively, if the biopsy indicates a result beyond periapical granuloma, cyst or fibrous connective tissue, a consultation with oral and maxillofacial surgery to determine the further treatment plan will be needed. Further studies and more cases are required to determine the outcome of this treatment approach.

**Table 1:** Patients’ Details and Treatment Outcomes

Case no.	Age (y)	Sex	Tooth no.	Size of lesion (mm)	Preoperative diagnosis	GTR	Pathological examination	Last follow-up(mo)	Healing assessment criteria	Treatment Outcomes
1	27	F	#8	8 × 12 × 10	Periapical periodontitis	N	Periapical cyst	54	2D and 3D	Complete healing(2D) Limited healing(3D)
2	36	F	#29	8 × 14 × 15	Periapical periodontitis	Y	Periapical granuloma	12	2D	Complete healing
3	22	F	#9	15 × 10 × 15	Periapical periodontitis	Y	Periapical cyst	27	2D	Complete healing
4	21	F	#29	13 × 10 × 12	Periapical periodontitis	N	Periapical cyst	12	2D	Complete healing
5	22	F	#9	12 × 10 × 12	Periapical periodontitis	N	Periapical cyst	30	2D	Complete healing
6	30	F	#8	5 × 8 × 12	Periapical periodontitis	N	Periapical cyst	44	2D	Complete healing
7	25	M	#29	7 × 14 × 10	Periapical periodontitis	N	Periapical granuloma	24	2D	Complete healing
8	36	F	#19	18 × 11 × 11	Periapical periodontitis	N	Periapical granuloma	48	2D and 3D	Complete healing(2D) Limited healing(3D)

No, number; mm, millimeter; GTR, guided tissue regeneration; mo, month; F, female; M, male; N, no; Y, yes; 2D, 2 dimensions; 3D, 3 dimensions;

## Data Availability

The datasets used and/or analysed during the current study are available from. the corresponding author on reasonable request.

## Abbreviations

EMS: Endodontic microsurgery
RCT: Root canal treatment
ReRCT: Root canal retreatment
PAR: Periapical radiograph
CBCT: Cone-beam computed tomography
GTR: Guided tissue regeneration
no: Number
mm: Millimeter
mo: Month
F: Female
M: male
N: No
Y: Yes
2D: 2 dimensions
3D: 3 dimensions

## References

[1] Monaghan L, Jadun S, Darcey J (2019). Endodontic microsurgery. Part one: diagnosis, patient selection and prognoses. Br Dent J.

[2] Lin LM, Gaengler P, Langeland K (1996). Periradicular curettage. Int Endod J.

[3] Kim S, Kratchman S (2006). Modern endodontic surgery concepts and practice: a review. J Endod.

[4] Nesari R, Kratchman S, Saad M, Kohli MR (2020). Selective curettage: a conservative Microsurgical Approach to treating large and complicated lesions. J Endod.

[5] Chi CS, Andrade DB, Kim SG, Solomon CS (2015). Guided tissue regeneration in endodontic surgery by using a bioactive resorbable membrane. J Endod.

[6] Taschieri S, Del Fabbro M, Testori T, Weinstein R (2007). Efficacy of xenogeneic bone grafting with guided tissue regeneration in the management of bone defects after surgical endodontics. J Oral Maxillofac Surg.

[7] Molven O, Halse A, Grung B (1987). Observer strategy and the radiographic classification of healing after endodontic surgery. Int J Oral Maxillofac Surg.

[8] Schloss T, Sonntag D, Kohli MR, Setzer FC (2017). A comparison of 2- and 3-dimensional Healing Assessment after endodontic surgery using cone-beam computed Tomographic volumes or periapical radiographs. J Endod.

[9] Kang S, Ha SW, Kim U, Kim S, Kim E. A one-year Radiographic Healing Assessment after Endodontic Microsurgery using Cone-Beam Computed Tomographic Scans. J Clin Med. 2020;9(11). 10.3390/jcm9113714 PMC7699244 33228002

[10] Nair PN (2006). On the causes of persistent apical periodontitis: a review. Int Endod J.

[11] Karamifar K, Tondari A, Saghiri MA (2020). Endodontic Periapical Lesion: an overview on the etiology, diagnosis and current treatment modalities. Eur Endod J.

[12] Siqueira JF, Rôças IN, Ricucci D, Hülsmann M (2014). Causes and management of post-treatment apical periodontitis. Br Dent J.

[13] Siqueira JF, Rôças IN (2008). Clinical implications and microbiology of bacterial persistence after treatment procedures. J Endod.

[14] Pinto D, Marques A, Pereira JF, Palma PJ, Santos JM (2020). Long-term prognosis of endodontic Microsurgery-A systematic review and Meta-analysis. Med (Kaunas).

[15] Setzer FC, Shah SB, Kohli MR, Karabucak B, Kim S (2010). Outcome of endodontic surgery: a meta-analysis of the literature–part 1: comparison of traditional root-end surgery and endodontic microsurgery. J Endod.

[16] Setzer FC, Kohli MR, Shah SB, Karabucak B, Kim S (2012). Outcome of endodontic surgery: a meta-analysis of the literature–part 2: comparison of endodontic microsurgical techniques with and without the use of higher magnification. J Endod.

[17] Floratos S, Kim S (2017). Modern endodontic microsurgery concepts: a clinical update. Dent Clin North Am.

[18] Kim E, Song JS, Jung IY, Lee SJ, Kim S (2008). Prospective clinical study evaluating endodontic microsurgery outcomes for cases with lesions of endodontic origin compared with cases with lesions of combined periodontal-endodontic origin. J Endod.

[19] Fish EW (1939). Bone infection. J Am Dent Assoc.

[20] Carrillo C, Penarrocha M, Bagan JV, Vera F (2008). Relationship between histological diagnosis and evolution of 70 periapical lesions at 12 months, treated by periapical surgery. J Oral Maxillofac Surg.

[21] Nair PN (2004). Pathogenesis of apical periodontitis and the causes of endodontic failures. Crit Rev Oral Biol Med.

[22] Ten Cate AR (1972). The epithelial cell rests of Malassez and the genesis of the dental cyst. Oral Surg Oral Med Oral Pathol.

[23] Johnson NR, Gannon OM, Savage NW, Batstone MD (2014). Frequency of odontogenic cysts and tumors: a systematic review. J Investig Clin Dent.

[24] Su YK, Wang J, Zhang TF, Zhang ZB (2019). Odontogenic tumors and odontogenic cysts: a clinical and pathological analysis of 4 181 cases. Zhonghua Kou Qiang Yi Xue Za Zhi.

[25] Natkin E, Oswald RJ, Carnes LI (1984). The relationship of lesion size to diagnosis, incidence, and treatment of periapical cysts and granulomas. Oral Surg Oral Med Oral Pathol.

[26] Sullivan M, Gallagher G, Noonan V (2016). The root of the problem: occurrence of typical and atypical periapical pathoses. J Am Dent Assoc.

[27] Ramachandran Nair PN, Pajarola G, Schroeder HE (1996). Types and incidence of human periapical lesions obtained with extracted teeth. Oral Surg Oral Med Oral Pathol Oral Radiol Endod.

[28] Ricucci D, Rôças IN, Hernández S, Siqueira JF (2020). Jr. “True” Versus “Bay” apical cysts: clinical, Radiographic, histopathologic, and histobacteriologic features. J Endod.

[29] Nair PN (1998). New perspectives on radicular cysts: do they heal?. Int Endod J.

[30] Nair PN, Sjögren U, Schumacher E, Sundqvist G (1993). Radicular cyst affecting a root-filled human tooth: a long-term post-treatment follow-up. Int Endod J.

[31] Trope M, Pettigrew J, Petras J, Barnett F, Tronstad L (1989). Differentiation of radicular cyst and granulomas using computerized tomography. Endod Dent Traumatol.

[32] De Rosa CS, Bergamini ML, Palmieri M, Sarmento DJS, de Carvalho MO, Ricardo ALF (2020). Differentiation of periapical granuloma from radicular cyst using cone beam computed tomography images texture analysis. Heliyon.

[33] Pitcher B, Alaqla A, Noujeim M, Wealleans JA, Kotsakis G, Chrepa V (2017). Binary decision trees for Preoperative Periapical Cyst Screening using cone-beam computed Tomography. J Endod.

[34] Lizio G, Salizzoni E, Coe M, Gatto MR, Asioli S, Balbi T (2018). Differential diagnosis between a granuloma and radicular cyst: effectiveness of magnetic resonance imaging. Int Endod J.

[35] Santos Soares SM, Brito-Júnior M, de Souza FK, Zastrow EV, Cunha CO, Silveira FF (2016). Management of cyst-like Periapical lesions by Orthograde Decompression and Long-term Calcium Hydroxide/Chlorhexidine Intracanal Dressing: a Case Series. J Endod.

[36] Tian FC, Bergeron BE, Kalathingal S, Morris M, Wang XY, Niu LN (2019). Management of large Radicular Lesions using decompression: a Case Series and Review of the literature. J Endod.

[37] Bhaskar SN (1972). Nonsurgical resolution of radicular cysts. Oral Surg Oral Med Oral Pathol.

[38] Talpos-Niculescu RM, Popa M, Rusu LC, Pricop MO, Nica LM, Talpos-Niculescu S. Conservative Approach in the management of large Periapical Cyst-Like Lesions. A report of two cases. Med (Kaunas). 2021;57(5). 10.3390/medicina57050497 PMC8156608 34068934

[39] Lin LM, Huang GT, Rosenberg PA (2007). Proliferation of epithelial cell rests, formation of apical cysts, and regression of apical cysts after periapical wound healing. J Endod.

[40] Taschieri S, Testori T, Azzola F, Del Fabbro M, Valentini P (2008). Régénération tissulaire guidée en chirurgie endodontique [Guided-tissue regeneration in endodontic surgery]. Rev Stomatol Chir Maxillofac.

[41] Azim AA, Albanyan H, Azim KA, Piasecki L (2021). The Buffalo study: outcome and associated predictors in endodontic microsurgery- a cohort study. Int Endod J.

[42] Molven O, Halse A, Grung B (1996). Incomplete healing (scar tissue) after periapical surgery–radiographic findings 8 to 12 years after treatment. J Endod.

[43] Grung B, Molven O, Halse A (1990). Periapical surgery in a norwegian county hospital: follow-up findings of 477 teeth. J Endod.

[44] Bieszczad D, Wichlinski J, Kaczmarzyk T (2022). Factors affecting the success of Endodontic Microsurgery: A Cone-Beam Computed Tomography Study. J Clin Med.

[45] Stueland H, Ørstavik D, Handal T (2023). Treatment outcome of surgical and non-surgical endodontic retreatment of teeth with apical periodontitis. Int Endod J.

[46] Sakkas A, Winter K, Rath M, Mascha F, Pietzka S, Schramm A, Wilde F (2019). Factors influencing the long-term prognosis of root tip resected teeth. GMS Interdiscip Plast Reconstr Surg DGPW.

